# HIV-1 Subtypes B and C Unique Recombinant Forms (URFs) and Transmitted Drug Resistance Identified in the Western Cape Province, South Africa

**DOI:** 10.1371/journal.pone.0090845

**Published:** 2014-03-07

**Authors:** Graeme Brendon Jacobs, Eduan Wilkinson, Shahieda Isaacs, Georgina Spies, Tulio de Oliveira, Soraya Seedat, Susan Engelbrecht

**Affiliations:** 1 Division of Medical Virology, Stellenbosch University, Tygerberg, South Africa; 2 Africa Center for Health and Population Studies, University of KwaZulu-Natal, Mtubatuba, South Africa; 3 Department of Psychiatry, Stellenbosch University, Tygerberg, South Africa; 4 National Health Laboratory Services (NHLS), Tygerberg Coastal, South Africa; University of Florida, United States of America

## Abstract

South Africa has the largest worldwide HIV/AIDS population with 5.6 million people infected and at least 2 million people on antiretroviral therapy. The majority of these infections are caused by HIV-1 subtype C. Using genotyping methods we characterized HIV-1 subtypes of the *gag* p24 and *pol* PR and RT fragments, from a cohort of female participants in the Western Cape Province, South Africa. These participants were recruited as part of a study to assess the combined brain and behavioural effects of HIV and early childhood trauma. The partial HIV-1 *gag* and *pol* fragments of 84 participants were amplified by PCR and sequenced. Different online tools and manual phylogenetic analysis were used for HIV-1 subtyping. Online tools included: REGA HIV Subtyping tool version 3; Recombinant Identification Program (RIP); Context-based Modeling for Expeditious Typing (COMET); jumping profile Hidden Markov Models (jpHMM) webserver; and subtype classification using evolutionary algorithms (SCUEAL). HIV-1 subtype C predominates within the cohort with a prevalence of 93.8%. We also show, for the first time, the presence of circulating BC strains in at least 4.6% of our study cohort. In addition, we detected transmitted resistance associated mutations in 4.6% of analysed sequences. With tourism and migration rates to South Africa currently very high, we are detecting more and more HIV-1 URFs within our study populations. It is stil unclear what role these unique strains will play in terms of long term antiretroviral treatment and what challenges they will pose to vaccine development. Nevertheless, it remains vitally important to monitor the HIV-1 diversity in South Africa and worldwide as the face of the epidemic is continually changing.

## Introduction

HIV/AIDS is a major health problem in South Africa with approximately 5.6 million people infected with Human Immunodeficiency Virus type 1 (HIV-1) [1], the majority with HIV-1 subtype C. At least 2 million people are receiving antiretroviral therapy (ART) [2], the largest ART program world-wide. Although the epidemic in the country has stabilized during the last few years, an estimated 850 new infections still occur each day. In the latest annual antenatal survey it is estimated that approximately 29.5% of women between the ages of 15 and 49 are infected. High variations are also seen between the different provinces of South Africa. In the age group between 15 and 49 years, the Western Cape Province has the lowest prevalence (4.75%) and KwaZulu-Natal the highest (24.7%) [3].

HIV-1 is characterized by a high degree of genetic diversity and can be divided into four groups: M (major), O (outlier), N (non-M non-O) and P. Group M can further be divided into 9 subtypes (A–D, F–H, J, K) [4]. HIV-1 diversity is primarily caused by the fast replication cycle of the virus coupled with the high error prone rate of its reverse transcriptase (RT) enzyme [5]. Genetic recombination during the replication cycle of primate lentiviruses is a frequent event and also contributes to the global genetic variation of HIV-1 [6]. Intersubtype recombination is well documented for HIV-1 and at least 55 circulating recombinant forms (CRFs) and several unique recombinant forms (URFs) have been identified [http://www.hiv.lanl.gov/content/sequence/HIV/CRFs/CRFs.html]. The genetic variation of HIV-1 may influence diagnostic assays, viral replication capacity as well as antiretroviral therapy (ART) outcomes [6].

HIV-1 subtype C predominates worldwide with a prevalence of 52% [4] and is found in the Southern African region, the Indian sub-continent, with smaller subtype C epidemics in East African countries, Brazil and the southern provinces of the Peoples Republic of China [7]. HIV-1 subtype B is most prevalent in North America, Western Europe, Australia and Japan [7]. Many studies have shed light on the degree of sequence variation within the South African HIV/AIDS epidemic. Although the majority of people are infected with HIV-1 subtype C, with a minor subtype B epidemic [8], [9], other non-subtype C and URFs have sporadically been identified over the last few years [10]–[17].

In this study, we have characterized the HIV-1 subtypes in a cohort of female participants from the Western Cape Province in South Africa using different online tools and manual phylogenetic analysis. The genetic variability of HIV-1 in two genome regions were investigated in order to increase the chance of characterizing recombinants and/or non-C subtypes in South Africa. To the best of our knowledge this is the first time that HIV-1 subtype BC recombinants have been described in South Africa.

## Materials and Methods

### Ethics statement

This study was approved by the Health Research Ethics Committee (HREC) of Stellenbosch University (IRB0005239) and all study participants provided written informed consent for the collection of samples and subsequent analyses. The investigations also complies with the South Africa National Health Act No 612003 and abides by the ethical norms and principles for research as established by the Declaration of Helsinki, the South African Medical Research Council Guidelines as well as the Department of Health Guidelines.

### Study population and sample collection

EDTA blood samples and demographic information were collected, between 2008 and 2010, from 84 patients in the Western Cape Province. They were recruited from different HIV clinics and/or hospitals across the Cape Town Metropole, as well as the Paarl and Stellenbosch districts. The participants in the cohort were recruited as part of a study to assess the combined brain and behavioural effects of HIV and early childhood trauma, using neurocognitive and structural brain imaging assessments.

### HIV-1 Viral load and CD4+ cell counts

Viral loads were performed using the Abbott m2000sp and the Abbott m2000rt analysers (Abbott laboratories, Abbott Park, Illinois, USA). RNA was isolated from patient samples according to the manufacturer's instructions using the Abbott RealTime HIV-1 amplification reagent Kit. CD4 counts were done in conjunction with viral loads in order to determine the immune competence and viral burden for each patient. Analyses of cells were performed on the FACSCalibur flow cytometer in conjunction with the MultiSET V1.1.2 software (BD Biosciences, San Jose, CA, USA).

### PCR amplification of the partial gag p24 and pol regions

HIV-1 RNA was extracted from the plasma using the QIAamp Viral RNA kit (QIAGEN GmbH, Hilden, Germany) and the QIAcube automated extraction system, according to the manufactureŕs instructions and stored at −70°C until use. Two genomic regions of the HIV-1 genome were targeted for characterization: the *gag* p24 region (HXB2 nucleotides 1237–1721) and a segment of the *pol* gene, that includes the Protease (PR) and a partial segment of the Reverse Transcriptase (RT) region (HXB2 nucleotides 2082−3334), important for resistance analysis. PCR amplification and purification was done using previously described primers and methods [18], [19]. Briefly, cDNA synthesis and first round PCR amplification was done with the Access-RT PCR system (Promega, Wisconsin, USA), while second round nested PCR amplification was done with the GoTaq DNA polymerase system (Promega, Wisconsin, USA).

### DNA Sequencing

PCR products were sequenced using the BigDye Terminator v 3.1 Cycle Sequencing Ready Reaction Kit (Applied Biosystems, Foster City, CA, USA) and run on an ABI Prism 3130xl Genetic Analyzer (Applied Biosystems, Foster City, CA, USA), according to the manufactureŕs instructions. Both strands were sequenced using overlapping primers. Sequences were read and assembled into contigs using Sequencher v 5.1 (Gene Codes Corporation, Ann Arbor, MI, USA). All sequences were checked for quality assurance using the HIV-1 Sequence Quality Analysis tool (http://www.hiv.lanl.gov/content/sequence/QC/index.html) before further analyses.

### HIV-1 subtyping using online tools

All *gag* and *pol* sequences were preliminary subtyped with online HIV-1 subtyping tools using the default parameter settings: the REGA HIV Subtyping Tool Version 3. (http://www.bioafrica.mrc.ac.za/rega-genotype/html/subtypinghiv.html) [20], Recombinant Identification Program (RIP 3.0) (http://www.hiv.lanl.gov/content/sequence/RIP/RIP.html) [21], COMET HIV-1 (Context-based Modeling for Expeditious Typing) (http://www.comet.retrovirology.lu) and the jumping profile Hidden Markov Models (jpHMM) webserver http://www.jphmm.gobics.de/ [22], [23]. Subtype classification using evolutionary algorithms (SCUEAL) analysis was done using *pol* sequences only (http://www.datamonkey.org/dataupload_scueal.php) [24]. These tools were compared and the agreement (frequency of similar assignment) calculated.

### Phylogenetic and recombinant analysis

For phylogenetic analysis, the 2010 HIV-1 subtype reference sequence dataset was obtained from the Los Alamos National Laboratory (LANL) database (http://www.hiv.lanl.gov/content/sequence/NEWALIGN/align.html). Given the large number of HIV-1 subtype C sequences, as well as the identification of possible BC recombinants in the subtyping analyses, this reference data set were supplemented with additional randomly selected subtype B and C reference sequences from LANL. Sequence alignments were constructed in Clustal X2 (http://www.clustal.org/clustal2/) [25] and edited in Se-Al v 2.0 (http://tree.bio.ed.ac.uk/software/seal/). All positions with less than 95% site coverage were manually eliminated. Although the GeneCutter program in the HIV-1 Sequence Quality Analysis tool can codon align sequences, each data set were manually edited until a perfect codon alignment was obtained.

Five different alignments were constructed: 1 for the *gag* p24 data set and 4 different alignments for the *pol* data. Due to miss matches in the *pol* sequence data set (some fragments were smaller than 500 bp, while others were larger than 1000 bp) the *pol* data set were split into 4 based on fragment length and genomic region (PR or RT). The first *pol* data set is only 385 bp long (2250–2634 relative to HXB2) and encompassed a small portion of the 3′ terminal end of the *gag* coding region the entire *protease* (PR) coding region and a small portion of the 5′ coding region of the *reverse transcriptase* (RT) coding region. The second *pol* data set is only 769 bp long (2530–3300 relative to HXB2) and included a small portion of the 5′ coding region of PR and a large section of the RT coding region of HIV-1. The third *pol* data set is 549 bp long (2244–2792 relative to HXB2) and includes a small portion of the 3′ terminal end of the *gag* coding region the entire PR coding region and a small section of the 5′ coding region of the RT coding region. The fourth *pol* data set is 977 bp long (2250–3326 relative to HXB2) and encompassed a small portion of the 3′ terminal end of the *gag* coding region the entire PR coding region and a large part of the 5′ coding region of the RT coding region.

Multiple alignments were used to infer phylogenies with the use of both Maximum likelihood (ML) [26] and Bayesian tree construction methods as implemented in phyML v 3.0 (http://www.atgc-montpellier.fr/phyml/) and MrBayes v 2.0 (http://mrbayes.sourceforge.net/download.php) [27] respectively. For each data set phylogenies were inferred under the “best fitting” model of nucleotide substitution, which was calculated in ModelTest v 2.0 [28], [29] and the use of the Akaike Information Criterion (AIC). For each data set the best fitting model was used to infer evolutionary relationships. For the ML tree topologies a total of 1000 bootstrap replicates were performed for each data set [30]. Bayesian phylogenetic trees were inferred following 10 million chains in the Markov Chain with four independent runs being performed. Inferred tree topologies were visually inspected in FigTree v 1.3.1 (http://tree.bio.ed.ac.uk/software/figtree/).

### Drug Resistance analysis

Both the PR and RT fragments were submitted to the South African mirror of the Stanford University HIV Drug Resistance database [31] for analysis with HIVdb, the genotypic resistance interpretation algorithm version 6.1.1 (http://hivdb.stanford.edu/). The Calibrated Population Resistance (CPR) Tool Version 6 (http://cpr.stanford.edu/cpr.cgi ) was used to analyze our dataset for proportions with surveillance drug resistance mutations (SDRMs). Drug resistance mutations were scored as either major or minor mutations.

## Results

### Patient demographics

**Figure 1:**
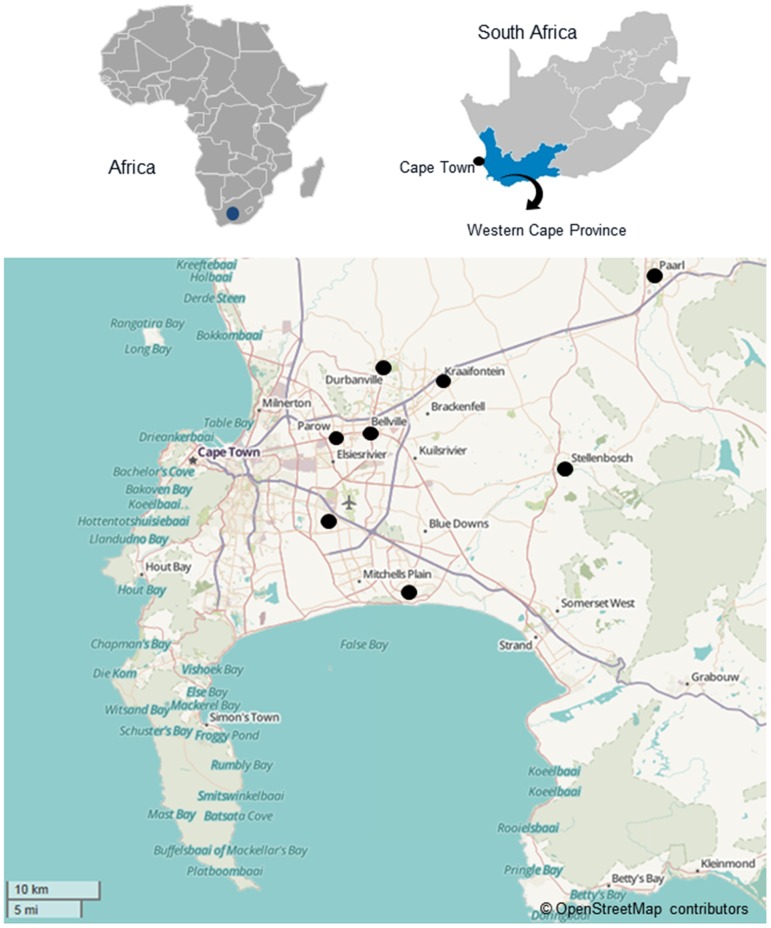
Map of the Western Cape region of South Africa, with the origin of the patients indicated. A total of 84 patient samples were obtained from the Cape Town Metropole: (Bellville, n = 15; Durbanville, n = 4; Khayelitsha, n = 35; Kraaifontein, n = 1; Mfuleni, n = 24; and Parow, n = 1). One patient came from the Paarl region and two patients came from the Stellenbosch region (**Figure 1**).

The patient demographics and clinical laboratory results are summarized in **Table S1**. Briefly, the cohort consisted of 84 female participants mostly from the Cape Town Metropole (Bellville, Durbanville, Khayelitsha, Kraaifontein, Mfuleni and Parow). One patient came from the Paarl and two patients came from the Stellenbosch district regions (**Figure 1**).

The majority (96.42%) of participants were African, with 3 participants (3.57%) of mixed race origin. The mean age of the cohort was 32.15 years (SD = 6.53) and ranged from 21 to 50 years. The CD4 lymphocyte count ranged from 35 to 1529, with a mean of 375.29 (SD = 267.02). The HIV viral load ranged from below the detectable limit to 3200000, with a mean of 111513.03 (SD = 416654.5).

### PCR amplification

PCR amplification and sequencing of the *gag* p24 region from the patient RNA of 84 patient samples yielded 67 (79.76%) positive samples, In addition 65 (75.58%) samples were also amplified and used for sequence analyses of the *pol* PR and RT fragments. In total 73 (86.9%) of cohort samples were successfully amplified for at least one region. The majority of patients from whom PCR products could not be amplified, had low viral loads (**Table S2**).

### Subtyping using REGA, jpHMM, RIP, COMET and SCUEAL online subtyping tools

In total, 67 sequences were used for the *gag* subtype assignment. The average size of the *gag* sequences were 433 bp. Using REGA 3.0 classification rules, 61 sequences (91.04%) were classified as subtype C. Six sequences (PM003-08, CS006-08, FG023-08, LM081-09, AQ123-10 and ZN124-10) were assigned with “check the report”. Analysis of the reports showed that 2 of the sequences (PM003-08, FG023-08) were assigned as subtype C, clustering within the pure subtype cluster with bootstrap support (> 70%). The other 4 sequences (CS006-08, LM081-09, AQ123-10 and ZN124-10) were also assigned as subtype C with bootstrap support (>70%); however they clustered outside the subtype C reference dataset (**Table S3**). RIP 3.0 could not characterize CD0777-08 and FG023-08, while the jpHMM program classified PT049-09 *gag* p24 as subtype D, but with a low posterior probability (0.6). With COMET all sequences were classified as subtype C.

With the 65 *pol* sequences analysed, with an average length of 917 bp (ranging from 391 to 1199 bp), 61 (93.85%) were classified as HIV-1 subtype C, whereas 3 (4.62%) sequences were characterized as subtype B. One sequence (TB089-09) was clearly identified as a possible BC recombinant using REGA 3.0, jpHMM, COMET, SCUEAL and RIP 3.0 analysis. In addition, two of the subtype B *pol* sequences (PM014-08 and MN091-09) had corresponding subtype C *gag* p24 sequences, subsequently identifying these strains as BC recombinants, however without detecting breakpoints in the *pol* region.

There were 5 *pol* sequences (NM026-08, TB037-09, SN055-09, BM-07209, and ZN124-10) which could not be characterized by REGA 3.0. These sequences were short sequences (average of 688 bp) classified as “check the report”, which suggests that the user check the results before assignment. Once the results were checked, all of these sequences could be classified as subtype C with an average bootstrap of 97%. With the jpHMM tool, PM003-08 (C/F2 subtype) and SB067-09 (A2/B/C subtype) could not be clearly subtyped. The RIP 3.0 software was also unable to subtype the MN091-09 *pol* sequence. The Stanford database also gave discrepancies with the *pol* subtyping with samples HN113-10 (K, C subtype) and NN140-10 (A, C subtype).

With SCUEAL analysis for the 65 *pol* sequences (**Table S4**), 8 sequences (12.31%) could be characterized as having B, C recombinant sequences. An additional 8 subtype C sequences also showed intra-subtype recombinant breakpoints. Furthermore, 2 sequences (NK032-08 and NN140-10) had C, F1 inter-subtype breakpoints, while 2 other sequences (SB067-09 and ZN124-10) had C, G breakpoints. One sequence (1.54%) each of subtype recombinants A2, C and C, H were also identified by the SCUEAL report. However, for 9 of the 22 (40.91%) recombinant samples identified by SCUEAL, the confidence assignment for the interpretation was low. These results should be further analyzed using longer sequences and/or more gene fragments.

In summary, 3 unique BC recombinant strains (PM014-08, TB089-09 and MN091-09), comprising 4.62% of our data set, were identified with the online subtyping tools.

### Phylogenetic analysis and subtyping of the gag gene

Maximum Likelihood (ML) and Bayesian tree topologies were inferred from the *gag* p24 sequence alignment. The sequence length was 441 bp long and phylogenies were inferred with the GTR model of nucleotide substitution, as was determined by the AIC analyses in ModelTest, and with an estimated Gamma shape parameter.

**Figure 2:**
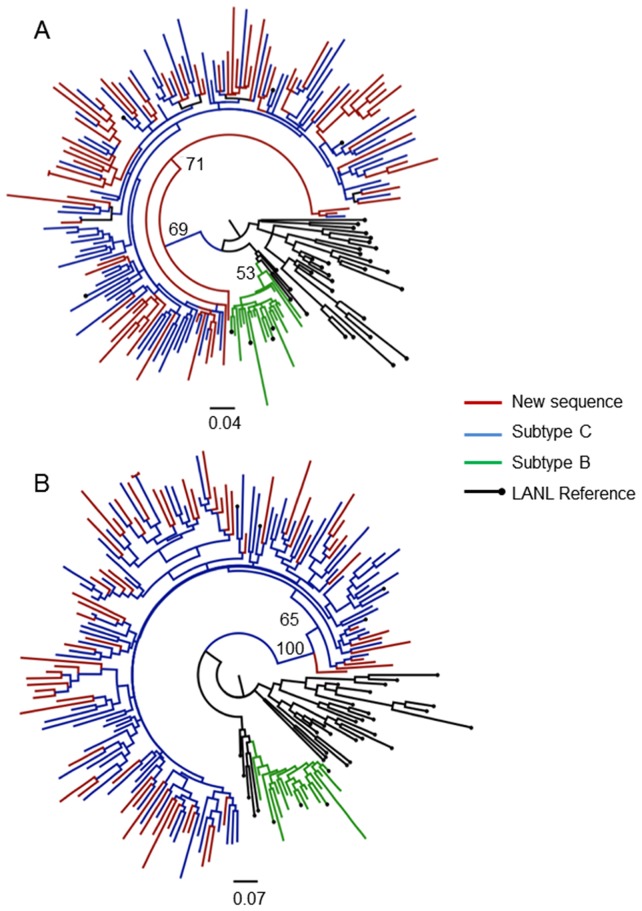
Phylogenetic analysis of *gag* p24 gene fragment. Evolutionary relationships were inferred with the use of the GTR model of nucleotide substitution and an estimated Gamma shape parameter. The alignment contained 203 taxa, including 68 of our own patient sequences, with an length of 441(1264–1704 relative to HXB2). Two different methods of tree inference were used: a Maximum Likelihood (A) and a Bayesian approach (B). The Maximum Likelihood tree topology was inferred in PhyML v 3.0 with 1000 bootstrap replicates. The Bayesian tree topology was inferred in MrBayes v 2.0 with a total of 10 million steps in the Markov Chain and four independent runs. Inferred trees were visually inspected in FigTree v 1.3.1. The most relavent bootstrap or posterior support values are indicated on the tree topology.

Both ML and Bayesian *gag* trees gave similar topology and branching patterns. Branches with a bootstrap value of 70% or greater were considered reliable, while in the Bayesian tree topology branches with a posterior support greater than 0.9 were considered trustworthy. The result of the ML and Bayesian phylogenetic analysis of the partial *gag* gene is shown in **Figure 2** **.**

All the *gag* sequences clustered within the subtype C clade in both the ML and Bayesian tree topologies. Only a single isolate did not cluster within the main subtype C clade, but clustered as an outlier to the subtype C clade in both the ML and Bayesian tree topologies. In the ML-tree topology isolate MH020-08 clustered as an outlier to the main subtype C clade with a bootstrap support value of 69%. The posterior support value for the interior branch connecting MH020-08 to the subtype C clade in the corresponding Bayesian tree topology was 1.0.

It should also be noted that two of the newly sequenced isolates in the *gag* data set clustered closely with one another with a small measure of genetic distance separating them. This is normally indicative of sequence contamination. However, these isolates were independently re-characterized in order to prevent for any potential sequence contamination. We were unable to amplify the *pol* fragment for both these sequences.

### Phylogenetic analysis and subtyping of the pol gene datasets

Four different *pol* sequence alignments (*pol*.dataset.1– *pol*.dataset.4) were used to infer Maximum Likelihood and Bayesian tree topologies. This was done in order to conserve sequence length in each alignment because of the varying fragment lengths of the newly sequenced isolates. ML and Bayesian phylogenies for all of the *pol* sequence alignments were inferred also with the use of the GTR model of nucleotide substitution and an estimated Gamma shape parameter.

Once again the results of the ML and Bayesian phylogenetic analyses of the partial *pol* gene gave similar tree topologies and branching patterns. All of the newly sequenced isolates were identified as subtype C isolates with the exception of four isolates. Bootstrap and probability values were supportive for all subtype C isolates, with the exception of the Bootstrap values in the ML-tree topology for the shorter *pol* data set. However, this may be a result due to the shorter fragment length of this alignment (385 bp).

**Figure 3:**
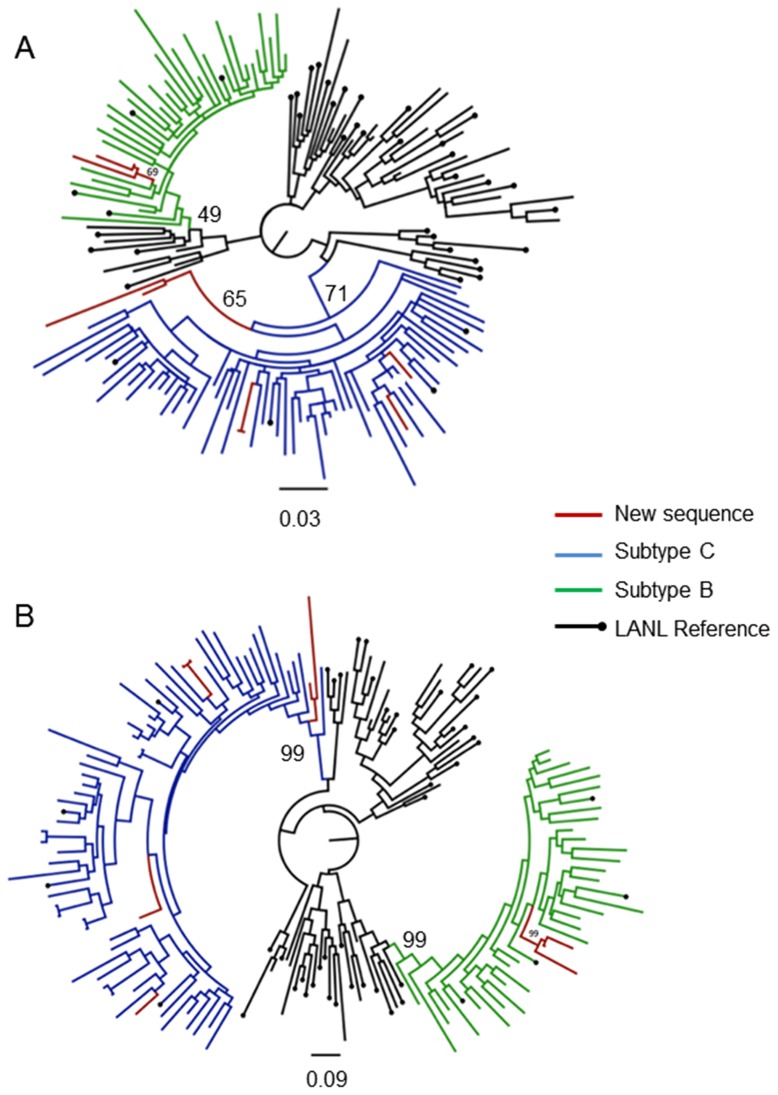
Phylogenetic analysis of the *pol* alignment (Dataset 1). The alignment contained 164 taxa, including nine sequences from our own data set, and with a total length of 385 bp (2250–2634 relative to HXB2). Evolutionary relationships were inferred with the use of the GTR model of nucleotide substitution and an estimated Gamma shape parameter. Two different methods of tree inference were used: a Maximum Likelihood (A) and a Bayesian approach (B). The Maximum Likelihood tree topology was inferred in PhyML v 3.0 with 1000 bootstrap replicates. The Bayesian tree topology was inferred in MrBayes v 2.0 with a total of 10 million steps in the Markov Chain and four independent runs. Inferred trees were visually inspected in FigTree v 1.3.1. The most relavent bootstrap or posterior support values are indicated on the tree topology.

Close examination of the various tree topologies that was inferred from the *pol*.dataset.1 alignment revealed that three isolates (PM014-08, AS050-09, and MN091-09) clustered within the subtype B clades for both the ML and Bayesian tree topologies (**Figure 3**). The branch support for this clustering however were below the accepted confidence levels in the ML-tree topology with an bootstrap support of only 38%, while the interior branch of the larger subtype B clade only had a branch support of 12%. However, in the corresponding Bayesian tree topologies the branch support for the internal branch of these three isolates were 1.0, while the posterior support for the internal branch of the larger subtype B clade that this cluster is nested in is 0.81.

**Figure 4:**
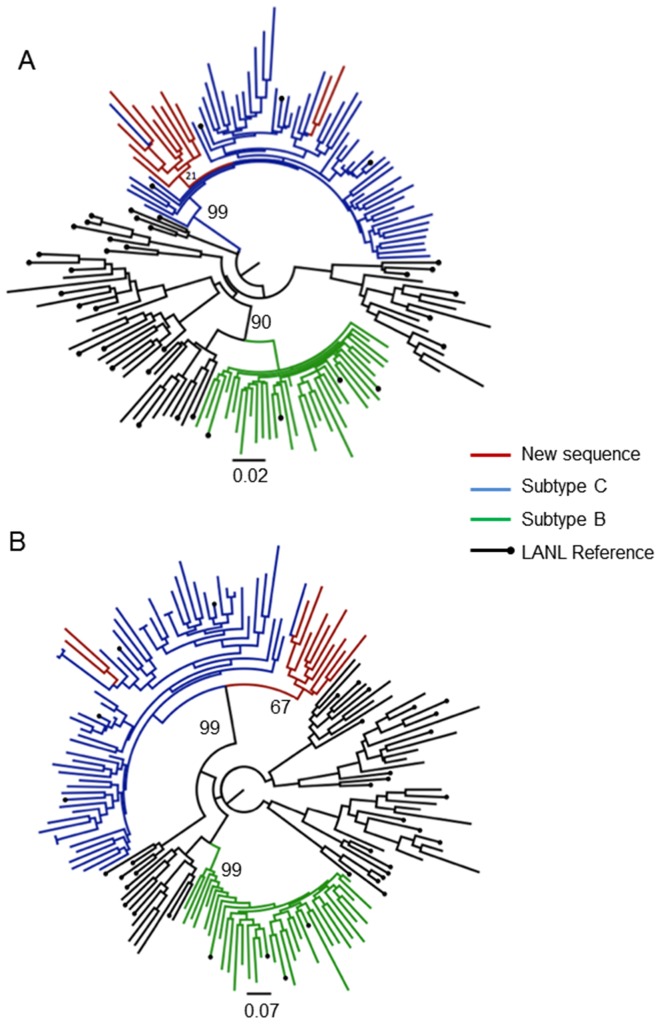
Phylogenetic analysis of the *pol* alignment (Dataset 2). Evolutionary relationships were inferred with the use of the GTR model of nucleotide substitution and an estimated Gamma shape parameter. The alignment contained 164 taxa, including 12 of our own patient sequences, with an length of 769 bp (2532–3300 relative to HXB2). Two different methods of tree inference were used: a Maximum Likelihood (A) and a Bayesian approach (B). The Maximum Likelihood tree topology was inferred in PhyML v 3.0 with 1000 bootstrap replicates. The Bayesian tree topology was inferred in MrBayes v 2.0 with a total of 10 million steps in the Markov Chain and four independent runs. Inferred trees were visually inspected in FigTree v 1.3.1. The most relavent bootstrap or posterior support values are indicated on the tree topology.

Inspection of the ML- and Bayesian tree topologies (**Figure 4**) that was inferred from the *pol*.dataset.2 alignment (769 bp) identified all of the 12 isolates as belonging to HIV-1 subtype C. However, it should also be noted that in the Bayesian inference from one of the *pol* data set (**Figure 4b**) coding in the reverse transcriptase region of HIV-1, all of the 12 isolates clustered along with one other South African subtype C reference strain as an outlier to the main subtype C clade. The posterior support for the internal branch of this cluster is 0.67, while the posterior for the internal branch of the larger subtype C clade is 1.0. However, in the corresponding ML-tree topology (**Figure 4a**) this clade clustered within the main subtype C clade with a bootstrap support of 99% for the internal branch of the larger clade. We can therefore conclude with confidence that our twelve isolates that were contained within this alignment belong to HIV-1 subtype C.

**Figure 5:**
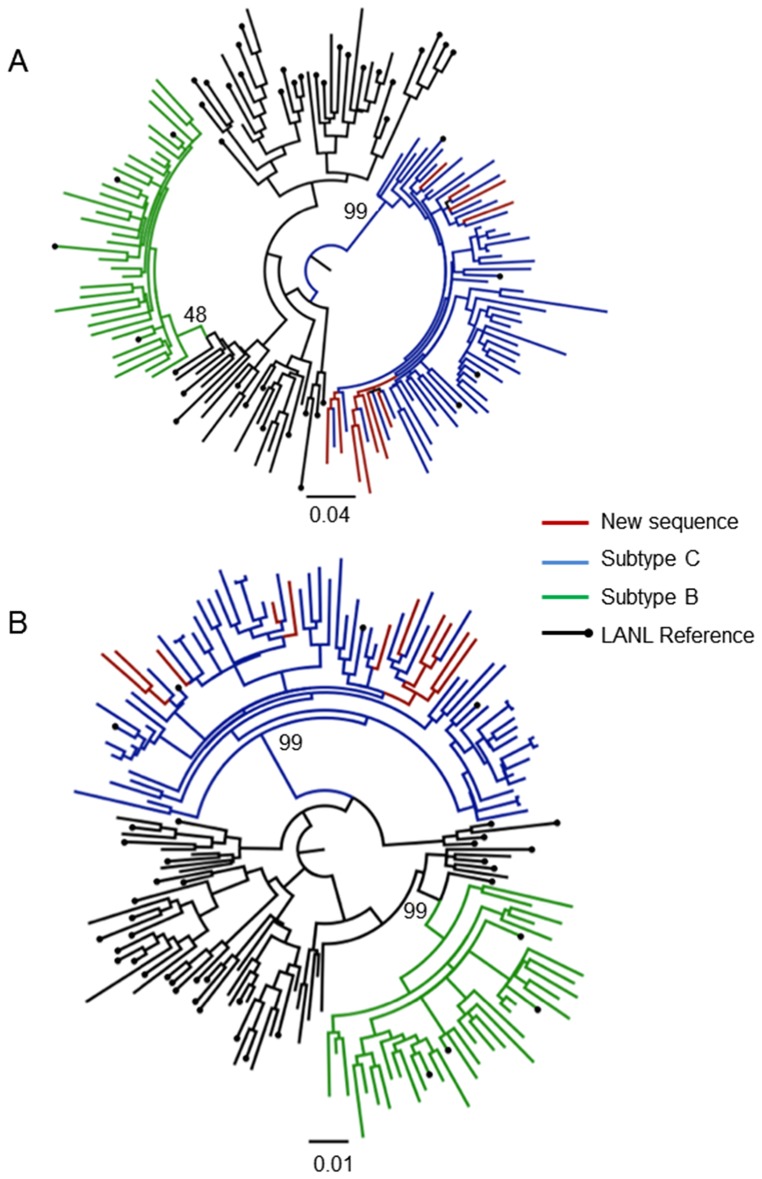
Phylogenetic analysis of the *pol* alignment (Dataset 3). Evolutionary relationships were inferred with the use of the GTR model of nucleotide substitution and an estimated Gamma shape parameter. The alignment contained 166 taxa, including 11 of our own patient sequences, with an length of 549 bp (2244–2792 relative to HXB2). Two different methods of tree inference were used: a Maximum Likelihood (A) and a Bayesian approach (B). The Maximum Likelihood tree topology was inferred in PhyML v 3.0 with 1000 bootstrap replicates. The Bayesian tree topology was inferred in MrBayes v 2.0 with a total of 10 million steps in the Markov Chain and four independent runs. Inferred trees were visually inspected in FigTree v 1.3.1. The most relavent bootstrap or posterior support values are indicated on the tree topology.

Furthermore, inspection of the ML- and Bayesian tree topologies that was inferred from the *pol*.dataset.3 alignment (**Figure 5**) identified all eleven of our patient samples as belonging to HIV-1 subtype C for this region of the genome with high bootstrap (99%) and posterior support (0.99).

**Figure 6:**
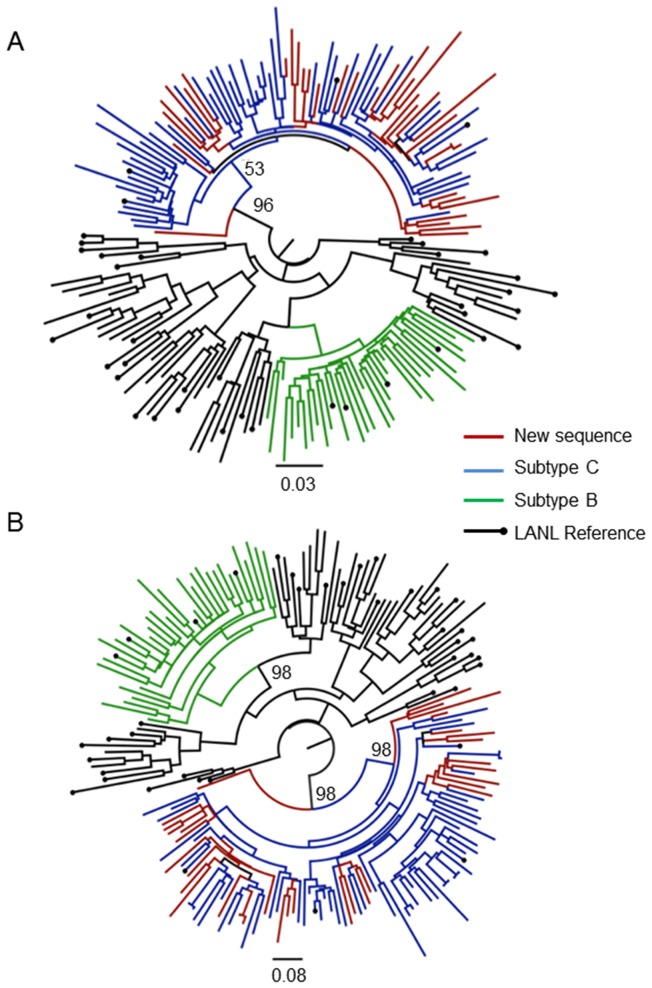
Phylogenetic analysis of the *pol* alignment (Dataset 4). Evolutionary relationships were inferred with the use of the GTR model of nucleotide substitution and an estimated Gamma shape parameter. The alignment contained 188 taxa, including 33 of our own patient sequences, with an length of 977(2250–3226 relative to HXB2). Two different methods of tree inference were used: a Maximum Likelihood (A) and a Bayesian approach (B). The Maximum Likelihood tree topology was inferred in PhyML v 3.0 with 1000 bootstrap replicates. The Bayesian tree topology was inferred in MrBayes v 2.0 with a total of 10 million steps in the Markov Chain and four independent runs. Inferred trees were visually inspected in FigTree v 1.3.1. The most relavent bootstrap or posterior support values are indicated on the tree topology.

Inspection of the ML- and Bayesian tree topologies that was inferred from the *pol*.dataset.4 alignment (**Figure 6**) identified all of the 33 isolates as subtype C with the exception of one patient sample TB089-09. The bootstrap support for the larger subtype C clade was 53% (**Figure 6a**), while the posterior support was 0.98 (**Figure 6b**). The clustering of TB089-09 as an outlier to the main subtype C clade with long branch lengths may be indicative of some recombination event, which must be further characterized using additional methods.

Therefore, the results of the online subtyping methods must be used in conjunction with the phylogenetic results in order to classify this isolate.

### Further Identification of possible recombinants

The jpHMM analysis was used to indicate the breakpoints of possible *pol* recombinants and these partial sequences were then used in NJ phylogenetic analysis for PM003-08, SB067-09 and TB089-09.

**Figure 7:**
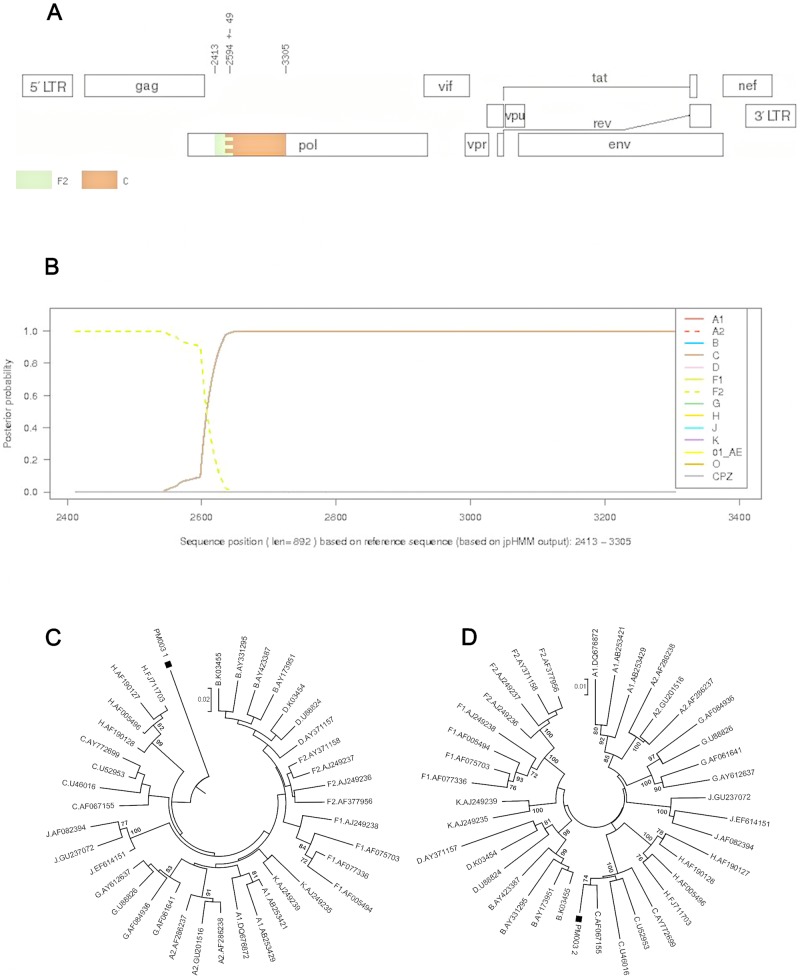
Subtyping analysis of PM003-08 using jpHMM and manual phylogenetic analysis. A). jpHMM result: A breakpoint is located at position 2594+/−49 (HXB2 numbering). B). Posterior probabilities of the subtypes at each sequence position (original sequence positions) calculated by jpHMM. Posterior probability values are indicated on the y axis and nucleotide positions in the alignment are shown on the x axis. C) and D). Phylogenetic analysis (NJ trees) of two fragments identified by the jpHMM breakpoint. C is a NJ tree of the unclassified fragment whose subtype classification was unresolved by posterior probability analysis and clustered with none of the HIV-1 reference subtypes from the Los Alamos HIV Sequence Database. D is a NJ tree of the subtype C region. Scale corresponds to nucleotide substitutions per site.

**Figure 8:**
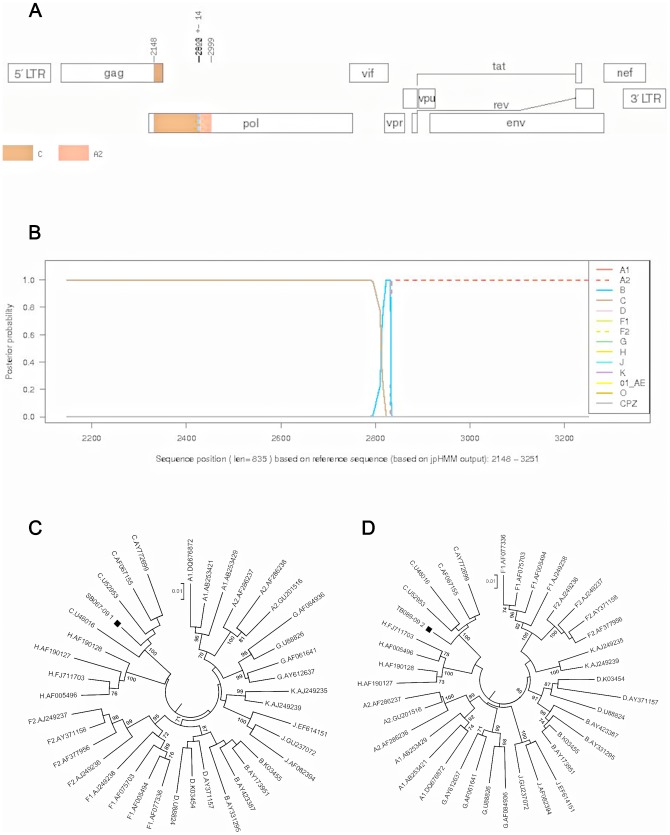
Subtyping analysis of SB067-09 using jpHMM and manual phylogenetic analysis. A). jpHMM result: A breakpoint is located at position 2820+/− 14 (HXB2 numbering). B). Posterior probabilities of the subtypes at each sequence position (original sequence positions) calculated by jpHMM. Posterior probability values are indicated on the y axis and nucleotide positions in the alignment are shown on the x axis. C) and D). Phylogenetic analysis (NJ trees) of two fragments identified by the jpHMM breakpoint.

**Figure 9:**
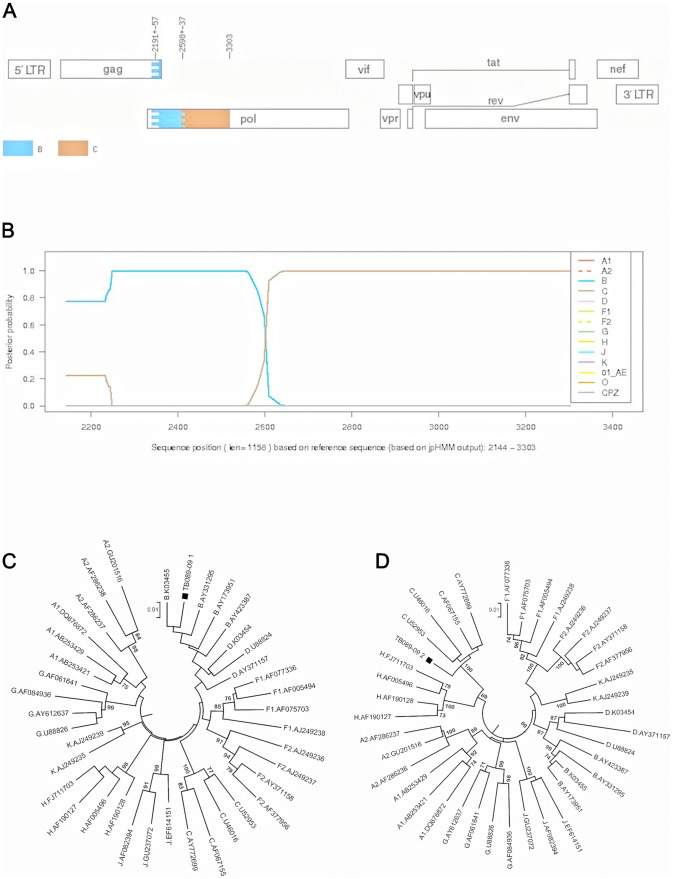
Subtyping analysis of TB089-89, a BC recombinant strain using jpHMM and manual phylogenetic analysis. A). jpHMM result: A breakpoint is located at position 2598+/−37 (HXB2 numbering). B). Posterior probabilities of the subtypes at each sequence position (original sequence positions) calculated by jpHMM. Posterior probability values are indicated on the y axis and nucleotide positions in the alignment are shown on the x axis. C) and D). Phylogenetic analysis (NJ trees) of two fragments identified by the jpHMM breakpoint. The breakpoint identified the majority of the PR as subtype B (HXB2 nucleotide 2148–2597) and the remaining RT as subtype C (HXB2 nucleotide 2597–3274).

PM003-08 (**Figure 7**) segment 1 was unclassified and segment 2 clustered with subtype C with 100% bootstrap support. SB067-09 (**Figure 8**) segment 1 clustered with subtype C with 100% bootstrap support, while segment 2 was an outlier to subtype C. TB089-09 (**Figure 9**) segment 1 clustered with subtype B with high bootstrap support of 86% and segment 2 clustered with subtype C with 100% bootstrap support. This sequence was also clearly identified as a possible BC recombinant using REGA 3.0, jpHMM, SCUEAL and RIP 3.0 analysis.

In addition SCUEAL identified 10 possible BC recombinants: NM026-08, TP029-09, NK032-08, NJ035-08, SN055-09, TB089-09, MN91-09, TM098-09, BD112-10, and ZN122-10. These results should be further analyzed using longer sequences and/or more gene fragments. SCUEAL uses a reference alignment, containing a larger number of reference strains per subtype, and are therefore more sensitive to uncover recombination breakpoints. Due to the small fragment length of some of our *pol* fragments it may be that other automated and manual methods that relies on reference alignments, may have missed small recombination events.

### Results of the subtyping agreement

The results of the subtyping agreement analyses between the various methods of subtyping and the manual phylogenetic analyses gave very compelling results (**Table 1**). From these results it would seem that manual phylogenetic inference to assign subtype still perform better when compared with most of the available online automated methods. Therefore, manual phylogenetic inference should still be regarded as the “gold standard” to assign HIV-1 subtypes. Furthermore, it would appear that there are broad agreement between REGA v 3.0, RIP3.0, Stanford, and jpHMM methods (**Table 1** **and** **2**). From the agreement analyses it would appear that SCUEAL performed the worst.

**Table 1:** Agreement between automated HIV-1 subtyping tools and manual phylogenetic analysis.

Agreement *pol* gene	REGA- check	RIP	jpHMM	SCUEAL	Stanford	Manual
**REGA V3**	92,31%	89,23%	89,23%	76,92%	87,69%	90,77%
**REGA- check**		96,92%	96,92%	80,00%	95,38%	98,46%
**RIP**			95,38%	78,46%	93,85%	95,38%
**jpHMM**				80,00%	95,38%	95,38%
**SCUEAL**					78,46%	78,46%
**Stanford**						93,85%
**Agreement ** ***gag*** ** gene**	**REGA- check**	**RIP**	**jpHMM**			**Manual**
**REGA V3**	92,54%	91,04%	91,04%			92,54%
**REGA -check**		97,01%	95,52%			97,01%
**RIP**			95,52%			97,01%
**jpHMM**						98,51%

RegaV3 results were presented in two formats, the first included only the automated results and the second (check) include checking the report for the result as suggested by the tool.

**Table 2:** Disagreement results between the different online tools in the *pol* Gene.

SeqID	Lenght	SCUEAL	Confidence Pure/recombinant		REGA V3	REGA V3 check	Bootstrapsubtype/inner/outer			RIP 3	jpHMM	Stanford	Manual	*gag pol* assignment
BM072-09	476	C			Check report	C	89	0	90	C	C	C	C	C/C
TB037-09	652	C			Check report	C	99	18	82	C	C	C	C	C/C
LM081-09	723	C			C	C	100	105	20	C	C	C	C	C/C
PS017-08	758	B,C	40.95	99.87	C	C	100	51	60	C	C	C	C	C/C
NK032-09	764	C,F1	93.86	99.95	C	C	100	40	64	C	C	C	C	C/C
TM098-09	766	C,H	40.43	99.99	C	C	100	39	58	C	C	C	C	C/C
ZN124-10	770	C,G	81.52	99.95	Check report	C	99	0	100	C	C	C	C	C/C
SN055-09	771	B,C	90.40	93.66	Check report	C	99	0	97	C	C	C	C	C/C
NJ035-09	775	B,C	56.98	100.00	C	C	100	36	64	C	C	C	C	C/C
NM026-08	775	B,C	75.00	96.13	Check report	C	100	16	81	C	C	C	C	C/C
ZN122-10	777	A2,C	48.50	99.99	C	C	100	49	58	C	C	C	C	C/C
SB067-09	835	C,G	74.88	75.06	C	C	100	48	59	A2,B,C	C,K	C,K	C	C/C
PM003-08	892	B,C	51.59	99.70	C	C	100	77	37	C	C,F2	C	C	C/C (U)
ZM126-10	1099	B,C	90.71	100.00	C	C	100	167	0	C	C	C	C	C/C
EF031-08	1111	C			C	C	100	92	28	C	C	C	C	C/C
GM103-09	1118	C			C	C	100	122	8	C	C	C	C	C/C
NN140-10	1154	C,F1	93.56	100.00	C	C	100	25	77	C	C	A,C	C	C/C (U)
AQ123-10	1157	C			C	C	100	32	72	C	C	C	C	C/C
TB089-09	1158	B,C	65.63	100.00	B,C	B,C	96	0	100	B,C	B,C	B,C	B	Recombinant
CD007-08	1176	B,C	64.28	66.99	C	C	100	169	0	C	C	C	C	C/C

### HIV-1 drug resistance

The majority (n = 58; 69.05%) of women were ART naïve, with 26 (30.95%) receiving treatment at the time of sample collection. We detected resistance-associated mutations (RAMs) in 6 of the 65 (9.23%) *pol* sequences analysed (**Table S5**). These include 3 samples (HN113-10, ND036-08 and ZN119-10) with the V82F PI mutation. The V82F mutation reduces susceptibility to most of the currently used PIs. One sample (NN140-10) had multiple RAMs, which include the NRTI mutation M184I and the well characterized NNRTI mutations K103N and Y181C. K103N was also found in sample NM026-08, while Y181C was also present in sample ZN122-10. M184I/V causes high-level resistance to lamivudine (3TC) and emtricitabine (FTC), while K103N causes high-level resistance to nevirapine (NVP) and efavirenz (EFV). Y181C also causes high-level resistance to NVP. Y181C also decreases susceptibility to etravirine (ETR), although in combination with other RAMs. Three patients (HN113-10, NN140-10 and NM026-08) were not on ART during the course of this study, thus transmitted resistance was present in 4.62% of the study population.

## Discussion

In this study we analysed the HIV-1 *gag* p24 as well as the *pol* PR and *pol* RT regions for sequence diversity within our study cohort. The *pol* region especially has become important for monitoring HIV-1 drug surveillance studies around the world [31]. However, *pol* analyses alone can not predict HIV-1 recombination accurately, therefore we also included the *gag* p24 analyses during our studies. The majority of our isolates were assigned to belong to HIV-1 subtype C. We detected 4 isolates, one subtype B isolates (AS052-09), and 3 BC recombinants (PM014-08, TB089-09 and MN091-09). Due to the inclusion of another subgenomic region into our analyses we were able to more accurately identify recombination within our data set, but only complete genome sequencing of each strain would give accurate results of the true number of recombinants present.

### HIV-1 diversity in South Africa

The early South African epidemic in the 1980's mirrored that of the epidemic in the industrialized world with HIV-1 subtype B circulating in the high risk groups of men who have sex with men (MSM) [9]. A minor subtype D epidemic was also present in the 1980's [9], [14]. However, since the early 1990's heterosexual transmission with HIV-1 subtype C has been the major route of HIV-1 transmission in the country [32].

In a previous study we showed that HIV-1 subtype C, predominantly spread via heterosexual transmission, accounts for approximately 95% of all infections in the Cape Town area [8]. South Africa has been dominated by the HIV-1 subtype C epidemic in the heterosexual population, with a minor subtype B epidemic found in the homosexual population [8], [9]. However, we have also lately seen an increase in the number of subtype B cases spread through heterosexual and mother-to-child transmission [8]. In this study, for the first time we show the crossover of the two epidemics with the emergence of new BC intersubtype recombinant strains, different to the strains circulating elsewhere, such as the CRF07 and CRF08 found in China [33]. BC Recombinant strains are also frequently found in Brazil where subtypes B, C and F1 are prevalent [34]. It is well known that the HIV-1 subtype C epidemic in Africa does not show a founder effect, but rather indicates that the subtype was introduced multiple times through mulitple lineages on the continent [35], [36]. It is therefore not surprising that recombination events between different HIV-1 strains are becoming more frequent. The high frequency of tourism and migration occurring could explain the rise of in the number of recombinant strains that have been identified. Wilkinson *et al* also previously highlighted the rise of the number of non-C strains that were being detected in Cape Town, South Africa [10].

### Online HIV-1 subtyping tools and phylogenetic analysis

With online tools becoming better models of prediction of recombination, it is easier to search for recombination breakpoints within our population and study cohorts. Phylogenetic analysis alone can not predict inter and intra-subtype breakpoints and has made online subtyping tools extremely useful within our population and study cohorts. There are a wide variety of online tools available, often using different algorithms to distinguish between the different HIV subtypes. However, we have noticed discrepencies between the various online subtyping tools that we have used in the study. The question that should be asked is how accurate are online subtyping tools and how long should a sequence segment be to give reliable information about that particular sequence. It has previously been reported that rapid subtyping tools often have low agreement in comparison with phylogenetic analysis, especially regarding non-B subtypes and HIV-1 recombinant forms [20], [21], [37]. SCUEAL has for example been shown to accurately type HIV strains not easily characterized by REGA version 2 [24]. Thus although online subtyping tools can be used as a quick guide for genotyping sequences, this should always be followed up with more accurate phylogenetic analyses.

### ART and drug resistance in South Africa

With 5.6 million people currently infected in South Africa, ART is steadily increasing. In this study and in previous studies we have shown that the rate of transmitted resistance is approximately 5% [38]. Therefore, it is a growing concern about the influence of resistance variants and other minor recombinant strains will have on treatment regimens. It is important to report only true resistance mutations and not to count sequence polymorpisms or minor mutations when reporting transmitted drug resistance. It is still unclear what role HIV-1 diversity will have on the impact of long term treatment [39]. In recent years newer strategies for vaccine development and ART have become more focussed on finding solutions against all major HIV-1 variants. Monitoring HIV-1 transmission patterns and genetic diversity across the world remains and important endeavour in our fight against HIV/AIDS.

### Sequence Data

The sequences analysed during the study have been deposited into GenBank and are available under the following accession numbers: *pol* sequences KF793121 to KF793185, *gag* sequences KF793054 to KF793120.

## Supporting Information

Table S1 **Patient demographic information of study cohort.** The cohort consisted of 84 female participants from different clinics in Cape Town, South Africa. The majority (n = 81; 96.42%) of participants were African, with 3 participants (3.57%) of mixed race origin. The mean age of the cohort was 31.5 years (SD = 6.53) and ranged from 21 to 50 years. The CD4 lymphocyte count ranged from 35 to 1529, with a mean of 630.48. The HIV viral load ranged from below the detectable limit to 3200000, with a mean of 101512.74.
(PDF)

Table S2 **Viral load of negative PCR patient samples.** There were 11 samples which could not be amplified with either the *gag* or *pol* fragments. Nine of theses samples had vary low viral loads, ranging from LDL to below 3000 copies/ml. The non-amplification of the 2 samples with higher viral loads, NG083-09 (19000) and AN141-10 (260000) is most likely due to the high variation between sequences of HIV-1.
(PDF)

Table S3 **Summary of** ***gag*** **p24 and** ***pol*** **PR and RT subtyping analysis.** We used the jpHMM, RIP 3.0, and REGA 3.0 online HIV subtyping tools to subtype our cohort sequences. In addition we also used SCUEAL analysis, currently only available for the *pol* region, to identify inter and intra subtype recombinant sequences. Phylogenetic analysis was done with MEGA 5.0. The summary indicates that the majority of samples were identified as HIV-1 subtype C, but a large number of recombinants were also identified, especially with SCUEAL analysis.
(PDF)

Table S4 **SCUEAL analysis of Unique Recombinant Forms (URFs).** With SCUEAL analysis as much as 22 (33.85%) of the 65 *pol* sequences were identified as having either inter or intra-subtype recombinant breakpoints. These include 8 (12.31%) subtype C sequences with intra-subtype recombination as well as 8 sequences (12.31%) with inter B, C recombinant sequences. Other recombinants identified by SCUEAL includes 2 (3.08%) sequences each of C, F1 and C, G recombinants as well as 1 (1.54%) sequence each of C, H and A2, C. However, 9 (40.91%) of these sequences had a confidence assignment of below 70%.
(PDF)

Table S5 **Sequences identified with SDRMs.** We identified RAMs in 6 (9.1%) of patient sequences. This includes resistance to 3TC, FTC, NVP and EFV.
(PDF)
