# Supplementary material for: HIV-1 Subtypes B and C Unique Recombinant Forms (URFs) and Transmitted Drug Resistance Identified in the Western Cape Province, South Africa
Source: PLoS One. 2014 Mar 7;9(3):e90845. doi: 10.1371/journal.pone.0090845 (PMC3946584; doi:10.1371/journal.pone.0090845)
Supplement: Table S1 — Patient demographic information of study cohort. The cohort consisted of 84 female participants from different clinics in Cape Town, South Africa. The majority (n = 81; 96.42%) of participants were African, with 3 participants (3.57%) of mixed race origin. The mean age of the cohort was 31.5 years (SD = 6.53) and ranged from 21 to 50 years. The CD4 lymphocyte count ranged from 35 to 1529, with a mean of 630.48. The HIV viral load ranged from below the detectable limit to 3200000, with a mean of 101512.74. (PDF) [file pone.0090845.s001.pdf]

Supplementary Table S1. Patient demographics of study cohort with viral load and CD4 data.

Patient ID	Sample date	Age	CD4+ cell count	VL	Ethnicity	ARV	Place / Town
KC002-08	2008/09/04	38	232	2000	MR	No	Durbanville
PM003-08	2008/07/22	30	217	3600	African	No	Mfuleni
PM004-08	2008/07/23	27	ND	2800	African	No	Khayelitsha
TG005-08	2008/07/05	23	810	4000	African	No	Kraaifontein
CS006-08	2008/08/26	33	165	1800000	African	No	Bellville
CD007-08	2008/09/02	35	ND	6200	African	No	Paarl
ZA008-08	2008/09/05	27	575	180	African	No	Mfuleni
YS010-08	2008/09/11	23	699	12000	African	No	Bellville
SR012-08	2008/09/19	35	390	190	African	No	Mfuleni
NM013-08	2008/09/23	25	886	1800	African	No	Mfuleni
PM014-08	2008/09/26	23	400	3400	African	No	Mfuleni
LN015-08	2009/09/25	33	579	LDL	African	No	Mfuleni
VG016-08	2008/10/08	27	538	51	African	No	Mfuleni
PS017-08	2008/10/07	38	600	50	African	No	Mfuleni
ST018-08	2008/10/09	21	628	7900	African	No	Mfuleni
MN019-08	2008/10/10	24	456	6500	African	No	Mfuleni
MH020-08	2008/10/29	23	257	98000	MR	NA	Bellville South
ND021-08	2008/10/30	27	360	1900	African	No	Mfuleni
FG023-08	2008/11/14	35	160	29000	African	No	Bellville
SM024-08	2008/11/19	37	121	7500	African	No	Bellville South
SK025-08	2008/11/19	36	680	LDL	African	No	Parow
NM026-08	2008/11/20	33	392	21000	African	No	Parow
WJ027-08	2008/11/21	30	320	52000	African	No	Khayelitsha
BM028-08	2008/11/21	43	256	180000	African	No	Khayelitsha
TP029-08	2008/11/14	33	690	2000	African	No	Khayelitsha
EF031-08	2008/12/03	34	268	1800	MR	No	Bellville South
NK032-08	2008/12/03	32	780	920	African	No	Khayelitsha
NY033_08	2008/12/03	29	300	830	African	Yes	Khayelitsha
XM034-08	2008/12/11	32	156	LDL	African	Yes	Bellville
NJ035-08	2008/12/11	50	240	1200	African	Yes	Durbanville
ND036-08	2008/12/12	39	340	690000	African	Yes	Khayelitsha
TB037-09	2009/01/30	29	374	1600	African	Yes	Khayelitsha
PM038-09	2009/02/04	26	270	200	African	Yes	Durbanville
NJ039-09	2009/02/05	25	416	2500	African	Yes	Khayelitsha
ZP040-09	2009/02/06	39	148	320000	African	Yes	Durbanville
GN041-09	2009/02/17	45	458	20000	African	Yes	Khayelitsha
TM042-09	2009/02/17	34	519	270	African	Yes	Khayelitsha
MD045-09	2009/03/05	27	845	7400	African	Yes	Mfuleni
JK047-09	2009/03/06	28	304	2400	African	Yes	Khayelitsha
NP048-09	2009/03/12	30	489	1100	African	Yes	Khayelitsha
PT049-09	2009/03/13	30	157	84000	African	No	Khayelitsha
AS052-09	2009/03/24	47	229	LDL	African	No	Mfuleni
SN055-09	2009/04/07	23	354	16000	African	No	Mfuleni
NF056-09	2009/04/08	32	321	16000	African	No	Khayelitsha
SB067-09	2009/04/30	44	400	1000	African	No	Khayelitsha
PM068-09	2009/04/30	27	777	2600	African	No	Mfuleni
BM072-09	2009/05/19	41	589	LDL	African	No	Mfuleni
LM081-09	2009/07/16	30	195	7900	African	No	Stellenbosch

NM082-09	2009/07/29	28	377	37000	African	No	Khayelitsha
NG083-09	2009/07/30	34	414	19000	African	No	Khayelitsha
RG084-09	2009/07/31	35	491	570	African	No	Stellenbosch
NN087-09	2009/08/18	27	163	160000	African	No	Khayelitsha
TG088-09	2009/08/24	34	364	8600	African	No	Khayelitsha
TB089-09	2009/08/02	45	535	1800	African	No	Khayelitsha
NM090-09	2009/09/09	33	72	180000	African	No	Khayelitsha
MN091-09	2009/09/09	25	478	22000	African	No	Khayelitsha
NS092-09	2009/09/10	26	1053	78	African	No	Khayelitsha
PK093-09	2009/09/14	39	587	27	African	No	Mfuleni
TM098-09	2009/10/01	26	690	61000	African	No	Khayelitsha
MT100-09	2009/10/21	41	1529	12000	African	No	Khayelitsha
CM103-09	2009/11/11	43	218	100000	African	No	Mfuleni
AZ111-10	2010/01/22	26	490	8000	African	No	Mfuleni
BD112-10	2010/01/21	36	508	20000	African	No	Khayelitsha
HN113-10	2010/01/25	38	275	5900	African	No	Khayelitsha
NM114-10	2010/01/25	48	572	45000	African	No	Khayelitsha
BL115-10	2010/01/27	25	586	7200	African	No	Mfuleni
PK116-10	2010/01/28	35	347	230000	African	No	Khayelitsha
NN117-10	2010/02/03	29	411	22000	African	No	Khayelitsha
NS118-10	2010/02/12	25	703	330000	African	Yes	Mfuleni
ZN119-10	2010/02/25	34	333	11000	African	Yes	Khayelitsha
ZM120-10	2010/03/05	35	206	220	African	Yes	Bellville
NS121-10	2010/03/11	34	111	210000	African	Yes	Mfuleni
ZN122-10	2010/03/11	25	668	350	African	Yes	Mfuleni
AQ123-10	2010/03/23	29	48	190000	African	Yes	Bellville
ZN124-10	2010/03/24	28	228	2400	African	Yes	Bellville
BM125-10	2010/03/25	34	35	150	African	Yes	Bellville
ZM126-10	2010/03/08	26	60	3200000	African	Yes	Mfuleni
VN127-10	2010/04/13	39	46	4800	African	Yes	Bellville
ET133-10	2010/05/03	35	162	120	African	Yes	Bellville
VZ134-10	2010/05/06	34	282	10	African	Yes	Bellville
LN135-10	2010/05/11	28	46	LDL	African	Yes	Bellville
BS139-10	2010/06/08	33	231	29000	African	No	Khayelitsha
NN140-10	2010/06/09	28	124	100000	African	No	Khayelitsha
AN141-10	2010/06/23	29	286	260000	African	No	Khayelitsha

*ND, Not determined; LDL, Lower than detection limit; MR, Mixed Race
