# Supplementary material for: HIV-1 Subtypes B and C Unique Recombinant Forms (URFs) and Transmitted Drug Resistance Identified in the Western Cape Province, South Africa
Source: PLoS One. 2014 Mar 7;9(3):e90845. doi: 10.1371/journal.pone.0090845 (PMC3946584; doi:10.1371/journal.pone.0090845)
Supplement: Table S2 — Viral load of negative PCR patient samples. There were 11 samples which could not be amplified with either the gag or pol fragments. Nine of theses samples had vary low viral loads, ranging from LDL to below 3000 copies/ml. The non-amplification of the 2 samples with higher viral loads, NG083-09 (19000) and AN141-10 (260000) is most likely due to the high variation between sequences of HIV-1. (PDF) [file pone.0090845.s002.pdf]

Supplementary Table S2. Viral load of negative PCR patient samples.

Patient ID	Viral load	*gag* subtype	*pol* subtype
KC002-08	2000	C	Negative
SR012-08	190	C	Negative
LN015-08	LDL	Negative	Negative
VG016-08	51	Negative	Negative
NY033-08	830	C	Negative
XM034-08	LDL	Negative	Negative
ND036-08	690000	C	Negative
JK047-09	2400	Negative	C
NP048-09	1100	Negative	C
AS052-09	LDL	Negative	B
NF056-09	16000	C	Negative
SB067-09	1000	Negative	C (U)
PM068-09	2600	Negative	Negative
BM072-09	LDL	Negative	C
NG083-09	19000	Negative	Negative
TG088-09	8600	C	Negative
NM090-09	180000	C	Negative
NS092-09	78	C	Negative
PK093-09	27	Negative	Negative
HN113-10	5900	Negative	C
ZM120-10	220	Negative	Negative
BM125-10	150	Negative	Negative
ET133-10	120	Negative	Negative
VZ134-10	10	Negative	Negative
AN141-10	260000	Negative	Negative

LDL, lower than detection limit; negative, PCR negative
