# Supplementary material for: HIV-1 Subtypes B and C Unique Recombinant Forms (URFs) and Transmitted Drug Resistance Identified in the Western Cape Province, South Africa
Source: PLoS One. 2014 Mar 7;9(3):e90845. doi: 10.1371/journal.pone.0090845 (PMC3946584; doi:10.1371/journal.pone.0090845)
Supplement: Table S3 — Summary of gag p24 and pol PR and RT subtyping analysis. We used the jpHMM, RIP 3.0, and REGA 3.0 online HIV subtyping tools to subtype our cohort sequences. In addition we also used SCUEAL analysis, currently only available for the pol region, to identify inter and intra subtype recombinant sequences. Phylogenetic analysis was done with MEGA 5.0. The summary indicates that the majority of samples were identified as HIV-1 subtype C, but a large number of recombinants were also identified, especially with SCUEAL analysis. (PDF) [file pone.0090845.s003.pdf]

Supplementary Table S3: Summary of *gag* p24 and *pol* PR and RTsubtyping analysis

	***gag* p24**					***pol* PR and RT**							
**Patient ID**	**REGA 3.0**	**RIP 3.0**	**jpHMM**	**MEGA 5.0**	**Subtype assigned**	**SCUEAL**	**REGA 3.0**	**RIP 3.0**	**jpHMM**	**Stanford**	**MEGA 5.0**	**Subtype assigned**	**Final *gag* / *pol***
KC002-08	C	C	C	C	C								C / NA
PM003-08	Check report	C	C	C	C	B, C recombinant	C	C	C, F2	C	C	C	C / C (U)
PM004-08	C	C	C	C	C	C	C	C	C	C	C	C	C / C
TG005-08	C	C	C	C	C	C	C	C	C	C	C	C	C / C
CS006-08	Check report	C	C	C	C	C	C	C	C	C	C	C	C / C
CD007-08	C	Undetermined	C	C	C	B, C recombinant	C	C	C	C	C	C	C / C
ZA008-08	C	C	C	C	C	C	C	C	C	C	C	C	C / C
YS010-08	C	C	C	C	C	C	C	C	C	C	C	C	C / C
SR012-08	C	C	C	C	C								C / NA
NM013-08	C	C	C	C	C	C	C	C	C	C	C	C	C / C
PM014-08	C	C	C	C	C	B	B	B	B	B	B	B	Recombinant
PS017-08	C	C	C	C	C	B, C recombinant	C	C	C	C	C	C	C / C
ST018-08	C	C	C	C	C	C	C	C	C	C	C	C	C / C
MN019-08	C	C	C	C	C	C	C	C	C	C	C	C	C / C
MH020-08	C	C	C	C	C	C	C	C	C	C	C	C	C / C
ND021-08	C	C	C	C	C								C / NA
FG023-08	Check report	Undetermined	C	C	C	C	C	C	C	C	C	C	C / C
SM024-08	C	C	C	C	C	C	C	C	C	C	C	C	C / C
SK025-08	C	C	C	C	C	C	C	C	C	C	C	C	C / C
NM026-08	C	C	C	C	C	B, C recombinant	Check report	C	C	C	C	C	C / C
WJ027-08	C	C	C	C	C	C	C	C	C	C	C	C	C / C
BM028-08	C	C	C	C	C	C	C	C	C	C	C	C	C / C
TP029-08	C	C	C	C	C								C / NA
EF031-08	C	C	C	C	C	C	C	C	C	C	C	C	C / C
NK032-08	C	C	C	C	C	C, F1 recombinant	C	C	C	C	C	C	C / C
NY033-08	C	C	C	C	C								C / NA
NJ035-08	C	C	C	C	C	B, C recombinant	C	C	C	C	C	C	C / C
ND036-08	C	C	C	C	C	C	C	C	C	C	C	C	C / C
TB037-09	C	C	C	C	C	C	Check report	C	C	C	C	C	C / C
PM038-09	C	C	C	C	C	C	C	C	C	C	C	C	C / C
NJ039-09	C	C	C	C	C	C	C	C	C	C	C	C	C / C
ZP040-09	C	C	C	C	C	C	C	C	C	C	C	C	C / C
GN041-09	C	C	C	C	C	C	C	C	C	C	C	C	C / C
TM042-09	C	C	C	C	C	C	C	C	C	C	C	C	C / C
MD045-09	C	C	C	C	C	C	C	C	C	C	C	C	C / C
JK047-09						C	C	C	C	C	C	C	NA / C
NP048-09						C	C	C	C	C	C	C	NA / C
PT049-09	C	C	D	C	C	C	C	C	C	C	C	C	C / C
AS052-09						B	B	B	B	B	B	B	NA / B
SN055-09	C	C	C	C	C	B,C recombinant	Check report	C	C	C	C	C	C / C
NF056-09	C	C	C	C	C								C / NA
SB067-09						C, G recombinant	C	A2, B, C	C, K	C,K	C	C	C / C
BM072-09						C	Check report	C	C	C	C	C	C / C
LM081-09	Check report	C	C	C	C	C	C	C	C	C	C	C	C / C
NM082-09	C	C	C	C	C	C	C	C	C	C	C	C	C / C
RG084-09	C	C	C	C	C	C	C	C	C	C	C	C	C / C
NN087-09	C	C	C	C	C	C	C	C	C	C	C	C	C / C
TG088-09	C	C	C	C	C								C / NA
TB089-09	C	C	C	C	C	B, C recombinant	B, C	B, C	B, C	B, C	B	B	Recombinant
NM090-09	C	C	C	C	C								C / NA
MN091-09	C	C	C	C	C	B	B	Undetermined	B	B	B	B	Recombinant
NS092-09	C	C	C	C	C								C / NA
TM098-09	C	C	C	C	C	C, H recombinant	C	C		C	C	C	C / C
MT100-09	C	C	C	C	C	C	C	C	C	C	C	C	C / C
CM103-09	C	C	C	C	C	C	C	C	C	C	C	C	C / C
AZ111-10	C	C	C	C	C	C	C	C	C	C	C	C	C / C
BD112-10	C	C	C	C	C	C	C	C	C	C	C	C	C / C
HN113-10						C	C	C	C	C, K	C	C	NA / C
NM114-10	C	C	C	C	C	C	C	C	C	C	C	C	C / C
BL115-10	C	C	C	C	C	C	C	C	C	C	C	C	C / C
PK116-10	C	C	C	C	C	C	C	C	C	C	C	C	C / C
NN117-10	C	C	C	C	C	C	C	C	C	C	C	C	C / C
NS118-10	C	C	C	C	C	C	C	C	C	C	C	C	C / C
ZN119-10	C	C	C	C	C	C	C	C	C	C	C	C	C / C
NS121-10	C	C	C	C	C	C	C	C	C	C	C	C	C / C
ZN122-10	C	C	C	C	C	A2, C recombinant	C	C	C	C	C	C	C / C
AQ123-10	Check report	C	C	C	C	C	C	C	C	C	C	C	C / C
ZN124-10	Check report	C	C	C	C	C, G recombinant	Check report	C	C	C	C	C	C / C
ZM126-10	C	C	C	C	C	B, C recombinant	C	C	C	C	B	B	C / C
VN127-10	C	C	C	C	C	C	C	C	C	C	C	C	C / C
LN135-10	C	C	C	C	C	C	C	C	C	C	C	C	C / C
NN138-10	C	C	C	C	C	C	C	C	C	C	C	C	C / C
BS139-10	C	C	C	C	C	C	C	C	C	C	C	C	C / C
NN140-10	C	C	C	C	C	C, F1 recombinant	C	C	C	A, C	C	C	C / C (U)
