# Supplementary material for: HIV-1 Subtypes B and C Unique Recombinant Forms (URFs) and Transmitted Drug Resistance Identified in the Western Cape Province, South Africa
Source: PLoS One. 2014 Mar 7;9(3):e90845. doi: 10.1371/journal.pone.0090845 (PMC3946584; doi:10.1371/journal.pone.0090845)
Supplement: Table S4 — SCUEAL analysis of Unique Recombinant Forms (URFs). With SCUEAL analysis as much as 22 (33.85%) of the 65 pol sequences were identified as having either inter or intra-subtype recombinant breakpoints. These include 8 (12.31%) subtype C sequences with intra-subtype recombination as well as 8 sequences (12.31%) with inter B, C recombinant sequences. Other recombinants identified by SCUEAL includes 2 (3.08%) sequences each of C, F1 and C, G recombinants as well as 1 (1.54%) sequence each of C, H and A2, C. However, 9 (40.91%) of these sequences had a confidence assignment of below 70%. (PDF) [file pone.0090845.s004.pdf]

Supplementary Table S4: SCUEAL analysis of Unique Recombinant Forms (URFs)

Name	Subtype	Expanded subtype	Confidence in Assignment	Support for recombination	Support for intra-subtype recombination	Breakpoints
PM003-08	B, C recombinant	CRF28,C,C inter-subtype recombinant	51.59	99.70	0.00	109 (108-110); 619 (615-623)
CD007-08	B, C recombinant	C,C,B inter-subtype recombinant	64.28	66.99	1.21	183 (169-197); 970 (969-971)
PS017-08	B, C recombinant	CRF28,C,C,C inter-subtype recombinant	40.95	99.87	0.00	109 (108-110); 221 (190-252); 621 (618-624)
SM024-08	C	C intra-subtype recombinant (1 breakpoints)	83.20	86.34	86.34	463 (346-580)
NM026-08	B, C recombinant	B,C inter-subtype recombinant	75.00	96.13	0.00	109 (102-116)
EF031-08	C	C intra-subtype recombinant (2 breakpoints)	48.57	64.57	64.57	551 (548-554); 782 (774-790)
NK032-08	C, F1 recombinant	F1,C inter-subtype recombinant	93.86	99.95	0.00	105 (88-122)
NJ035-08	B, C recombinant	B,C,C inter-subtype recombinant	56.98	100.00	0.00	125 (124-126); 358(349-367)
SN055-09	B, C recombinant	C,B,C inter-subtype recombinant	90.40	93.66	0.02	226 (225-227); 359 (347-371)
SB067-09	C, G recombinant	C,G,C inter-subtype recombinant	74.88	75.06	0.17	290 (290-290); 416(409-423)
RG084-09	C	C intra-subtype recombinant (2 breakpoints)	85.39	86.18	86.18	290 (280-300); 560 (548-572)
TB089-09	B, C recombinant	B,C,C,C inter-subtype recombinant	65.63	100.00	0.00	357 (356-358); 494 (493-495); 862 (855-869)
TM098-09	C, H recombinant	H,C inter-subtype recombinant	40.43	99.99	10.05	127 (109-145)
AZ111-10	C	C,CRF31 inter-subtype recombinant	93.28	97.85	0.01	263 (242-284)
NN117-10	C	C intra-subtype recombinant (1 breakpoints)	99.61	99.91	99.91	335 (211-459)
ZN119-10	C	C intra-subtype recombinant (2 breakpoints)	66.79	66.83	66.83	181 (153-209); 815 (180-1024)
ZN122-10	A2, C recombinant	A2,C inter-subtype recombinant	48.50	99.99	0.03	166 (143-189)
AQ123-10	C	C intra-subtype recombinant (2 breakpoints)	70.12	70.13	70.13	402 (382-422); 818 (811-825)
ZN124-10	C, G recombinant	C,C,G inter-subtype recombinant	81.52	99.95	0.00	546 (498-594); 655 (637-673)
ZM126-10	B, C recombinant	B,C inter-subtype recombinant	90.71	100.00	0.00	284 (272-296)
LN135-10	C	C intra-subtype recombinant (2 breakpoints)	70.42	70.49	70.45	105 (104-106); 458(437-479)
NN140-10	C ,F1 recombinant	C,F1,C inter-subtype recombinant	93.56	100.00	0.37	269 (256-282); 408 (407-409)
