# Supplementary material for: HIV-1 Subtypes B and C Unique Recombinant Forms (URFs) and Transmitted Drug Resistance Identified in the Western Cape Province, South Africa
Source: PLoS One. 2014 Mar 7;9(3):e90845. doi: 10.1371/journal.pone.0090845 (PMC3946584; doi:10.1371/journal.pone.0090845)
Supplement: Table S5 — Sequences identified with SDRMs. We identified RAMs in 6 (9.1%) of patient sequences. This includes resistance to 3TC, FTC, NVP and EFV. (PDF) [file pone.0090845.s005.pdf]

Supplementary Table S5: Sequences identified with SDRMs

**Patient ID**	**ARV**	**NRTI**	**NNRTI**	**PI**
NM026-08	No	None	**K103N**	NA
ND036-08	Yes	None	None	**V82F**
HN113-10	No	None	None	**V82F**
ZN119-10	Yes	None	None	**V82F**
ZN122-10	Yes	None	**Y181C**	NA
NN140-10	No	**M184I**	**K103N, Y181C**	None
